# The effects of continuous chest compression protocol on cardiopulmonary resuscitation (CPR) quality and fatigue

**DOI:** 10.1371/journal.pone.0353187

**Published:** 2026-07-13

**Authors:** Katie J. Lyman, Thomas A. Hanson, Jennifer A. Longo

**Affiliations:** 1 Archer College of Health and Human Services, Angelo State University, San Angelo, Texas, United States of America; 2 Department of Finance, Lacy School of Business, Butler University, Indianapolis, Indiana, United States of America; 3 School of Kinesiology, Physical Education, and Athletic Training, College of Education and Human Development, University of Maine, Orono, Maine, United States of America; UT Health San Antonio: The University of Texas Health Science Center at San Antonio, UNITED STATES OF AMERICA

## Abstract

**Aim:**

This study investigated how fatigue (measured both subjectively and objectively) and cardiopulmonary resuscitation (CPR) quality are impacted by various CPR protocols, principally comparing continuous chest compressions with intermittent rest periods.

**Methods:**

A sample of 23 participants (M = 22.3 years ± 3.8) was used in a cross-sectional design. Four compression:rest ratios were analyzed: 30:10, 50:10, 100:10, and continuous compressions (CC), with all participants performing CPR for 9 minutes in each condition. Subjective fatigue was measured using the Borg Rating of Perceived Exertion (RPE) at baseline, three, six, and nine minutes. Objective fatigue data were collected using the Parvo metabolic cart and heart rate (HR) monitors. Regression models of CPR performance quality (i.e., depth, recoil, and rate) investigated the effect of CPR protocol while controlling for participant variables. Fatigue (i.e., RPE, HR, and VO_2_) were compared across CPR protocols using repeated measure one-way ANOVA and post hoc Tukey’s honestly significant difference (HSD) tests.

**Results:**

Significant differences in performance were observed between CC and other protocols: 30 (p < .001), 50 (p = .009), and 100 (p = .031). Percent of full chest recoil was also significantly lower during CC than in 30, 50, and 100 conditions (all p < .001). Subjective fatigue increased significantly with longer compression ratios (p < .001).

**Conclusion:**

Higher compression ratios result in greater perceived fatigue and reduced CPR performance quality. Rescuer fatigue should be considered in CPR protocol design to optimize both rescuer effectiveness and patient outcomes.

## Introduction

Cardiac arrest remains a leading cause of mortality, with approximately 356,000 out-of-hospital cardiac arrests (OHCA) occurring annually in the United States and exhibiting a fatality rate of nearly 90% [1]. Prompt initiation of high-quality cardiopulmonary resuscitation (CPR) is essential for restoring circulation, particularly cerebral blood flow, and thereby improving survival rates [2]. The American Heart Association (AHA) currently endorses compression-only CPR for lay rescuers, arguing that it simplifies the intervention and increases bystander participation, based on the premise that some bystanders may feel uncomfortable providing ventilations, especially to strangers [2]. The AHA’s 2025 Guidelines further advocate continuous administration of chest compressions for untrained lay rescuers [2]. While these recommendations simplify the resuscitation process and enhance survival rates during OHCA, the evidence supporting the effectiveness of continuous chest compressions alone is limited [3,4]. Despite efforts to promote bystander intervention, OHCA survival rates remain suboptimal, and there is insufficient data to confirm that rescuers can consistently deliver high-quality resuscitation efforts through continuous chest compressions [5–7].

A critical determinant of CPR quality is fatigue such that researchers need to consider the physical and metabolic demands associated with performing CPR [8,9]. Continuous chest compressions can lead to rapid exhaustion, thus compromising the quality of care provided. Previous research suggests that performance degrades significantly and rapidly during compression-only CPR, and the decline in CPR quality is more pronounced than in traditional methods with ventilation pauses [5–7]. Some evidence suggests that incorporating short rest intervals can improve the efficacy of chest compressions [10,11].

Given that nearly 70% of out-of-hospital cardiac arrest (OHCA) incidents occur at home [12], often with only a single bystander present, it is crucial to establish CPR protocols that balance performance sustainability with effectiveness. Identifying optimal methods for high-quality care is especially vital since ambulance response times regularly exceed eight minutes [13]. Research-based recommendations can also inform modifications to CPR guidelines, which sometimes rely on expert consensus rather than robust scientific evidence [14].

While numerous studies confirm that compression-only CPR is preferable to no intervention, the evident decline in compression quality over time raises concerns regarding the overall effectiveness of CPR performed with continuous chest compressions [5,7]. The observed decline in performance quality of chest compressions is generally attributed to fatigue [6,10,15,16]. However, previous research has relied on various methods and measures of fatigue, including self-reported moment at which fatigue influences performance [6,16], physiological measurements [15], and self-reported fatigue on a visual analog scale [17]. The present study extends this previous work by adopting a multi-faceted approach to measuring fatigue, incorporating both self-reported (subjective) fatigue and objective physiological measurements.

Another factor that has resulted in discrepant findings among previous studies is the length of time participants perform CPR on a manikin. In some cases, a decrease in performance has been reported after only one minute [6], but comparisons of continuous chest compressions and traditional CPR methods sometimes vary as performance time increases [15]. The short durations of CPR measured in prior studies [6,15,17] may not adequately mirror real-world situations. Therefore, the present study supplements the extant literature by having participants perform continuous chest compressions for nearly 9 minutes.

The research protocol employed in this study evaluates the effectiveness of compression-only CPR across four protocols with different numbers of compressions between rest periods, while also assessing both objective and subjective measures of fatigue. This combination provides an enhanced understanding of the factors affecting CPR performance in emergent situations. The results provide evidence for potential improvements in CPR protocols that can enhance survival outcomes in OHCA scenarios by describing how performance and fatigue differ across the four CPR protocols.

## Materials and methods

This repeated measures, experimental study was approved by the university’s Institutional Review Board prior to participant recruitment. Written informed consent was obtained from all participants. A convenience sample of 23 participants (22.3 ± 3.8 years; 11 females and 12 males) volunteered to take part in this study from November 1, 2018, to May 1, 2019. Inclusion criteria targeted lay rescuers with current CPR certification. Exclusion criteria mandated that participants not be currently employed as an allied healthcare professional or currently enrolled in an allied health care curriculum at a university. Furthermore, participants must not have any musculoskeletal injuries or cardiac issues that would diminish the participant’s ability to perform high-quality CPR safely and effectively. Collectively, the inclusion and exclusion criteria imply a sample familiar with CPR but without professional experience to mimic the general bystander who might perform CPR in an emergency OHCA scenario.

Participants performed four compression-only CPR protocols over a two-day period. The four protocols included: 30 compressions followed by 10 seconds of rest (30:10), 50:10, 100:10, and as many compressions as possible with a 10-second rest based on participant preference, otherwise known as continuous compressions (CC) [10,17]. The justification for the chosen compression to break ratios is based on the research conducted by Min et al. [10] (100 & CC), Bjorshal et al. [17] (30 & 50), and the existing AHA recommendations [2] (30 and CC). Each of the protocols was completed for a total of eight minutes and 59 seconds (8:59), which represents the national average for an ambulance to arrive at an emergency in an urban environment [13]. Participants were randomized, and task order was counterbalanced. Participants performed two conditions on the first day, separated by a 15-minute break; the remaining two conditions were subsequently performed between 24 and 48 hours later. Each day of data collection included one smaller ratio (30:10, 50:10) and one larger ratio (100:10, CC).

On the first day of data collection, participants read and signed an informed consent form and provided demographic information, including gender, age, and years of CPR certification. Additionally, height and weight were measured to calculate BMI. Hand grip strength was also assessed using a hand grip dynamometer, with the recorded measurement being the average of three trials. Hand grip strength can be used as a predictor for overall strength and has been correlated to quality of chest compressions [18].

The researchers provided an overview of proper CPR administration, and the participants were allowed a one-minute CPR trial with deliberate feedback on the Resusci Anne® QCPR Manikin (Laerdal Medical AS, Stavanger, Norway), aligning with the familiarization protocols employed by Weber et al. [19] and Clark et al. [20], which ensure participants are acclimated to the equipment and improve the reliability of performance measurements. This medium-fidelity manikin records components of CPR performance including compression rate, compression depth, and chest recoil. The percent of chest compressions that met the AHA guidelines for compression rate, compression depth, and chest recoil was calculated as a measure of CPR quality.

Researchers then instructed participants about use of the Borg scale of perceived exertion (RPE) [21,22], which was assessed at baseline and every three minutes throughout each CPR protocol. One benefit of the simple RPE measurement is that it can be collected repeatedly and during physical activity [23]. Each participant wore a heart rate monitor (Polar electro, Kempele, FIN) and metabolic mask for the TrueOne 2400 Parvo Metabolic Cart (Parvo Medics, Sandy UT), which were used to collect data on heart rate and VO_2_. Measurements for these variables were recorded every 15 seconds throughout the trials. RPE is a subjective measure of fatigue, while heart rate and VO_2_ are included as objective measures of fatigue. Finally, participants completed the first two assigned CPR protocols on the Resusci Anne® manikin, duing which the participants were not allowed to view a clock and did not receive any feedback from the researchers during the trials. On the second day of data collection, participants completed the remaining two protocols.

All participants completed the four CPR protocols (30:10, 50:10, 100:10, and continuous compressions) in a randomized order to minimize order effects. Due to the nature of the task, participant blinding to protocol conditions was not possible; however, CPR performance metrics (compression depth, rate, and recoil) were collected objectively using a Laerdal Resusci Anne QCPR manikin, ensuring consistency and eliminating inter-rater variability. Objective fatigue measures (VO₂ and heart rate) were obtained via a calibrated Parvo Medics TrueOne 2400 metabolic cart and Polar heart rate monitors. No protocol deviations occurred, and all equipment was checked for calibration before each testing session.

Statistical analysis was conducted using the R Language, version 4.4.1 (R Foundation for Statistical Computing, Vienna, Austria), using the lme4 package [24] for mixed effects models and lmerTest package [25] for significance tests. Alpha was prespecified at 5% for all statistical tests. To investigate the three aspects of CPR performance (i.e., depth percent, recoil percent, and rate percent), mixed effects linear regression models were estimated, with a separate model for each of the CPR performance measures: depth, number of compressions, and compression rate. The models included gender, age, years certified, BMI, and grip strength as between-subjects factors and compression protocol as a within-subjects factor. Random intercepts for participants accounted for repeated measurements across the four conditions. within-subjects factor of compression protocol is the primary variable of interest in the model. To investigate differences in fatigue-related outcomes (i.e., RPE, VO_2_, and HR) across the four CPR protocols, we estimated two-way repeated measures ANOVA models with compression protocol (four levels) and time (four levels with measurements at start, 3, 6, and 9 minutes) as within-subjects factors using the afex package [26], which provides generalized eta-squared effect sizes. Greenhouse-Geiser corrected p-values are reported to control for violations of the sphericity assumption. Post-hoc comparisons employed Bonferroni-corrected pairwise comparisons with the emmeans package [27].

## Results

Participants had a mean age of 22.3 years (SD = 3.8), with ages ranging from 18.0 to 32.0 years. On average, they had been certified for 4.1 years (SD = 3.0), with a range of 0.0 to 14.0 years. The mean body mass index (BMI) was 25.5 kg/m² (SD = 5.3), ranging from 18.6 to 38.9 kg/m². Mean grip strength was 43.4 kg (SD = 12.0), with a range of 22.7 to 68.3 kg. The nature of the CPR protocols implies that participants will complete more compressions as the number in each set grows. For the 30:10 protocol, participants completed an average of 595.9 compressions (SD = 42.03), and this number increased predictably for the 50:10 protocol (M = 712.0, SD = 59.70), 100:10 (M = 865.1, SD = 87.94), and continuous compressions (M = 1013.3, SD = 110.11). Similarly, the chest compression fraction naturally increases with the number of compressions per set: for the 30:10 protocol (M = 63.2%, SD = 2.59%), 50:10 (M = 73.6%, SD = 2.21%), and 100:10 (M = 83.9%, SD = 1.63).

Each dependent measure of CPR performance (depth percent, recoil percent, and rate percent) was modeled with a mixed effects linear regression model to account for the repeated measures across CPR protocol as well as the following participant variables: gender, age, years of CPR certification, BMI, and grip strength. The sample size of 23 participants provided approximately 65% power for the mixed effects linear regression models in post-hoc analysis for a large effect size and an alpha level of 5%. Results of the first mixed effects linear regression model with depth percent as the dependent variable appear in Panel A of Table 1. The regression model was statistically significant in comparison to a null model (χ2 [8]=36.3, p < .001, Conditional R^2^ = 0.678). None of the between-subjects participant variables were statistically significant, but the repeated measure of CPR protocol was statistically significant (F[3, 66]=9.92, p < .001). The regression model employs continuous compressions as the baseline for comparison, and Table 1 shows that performance was better in all other conditions. Descriptive statistics of performance across the CPR protocols appear in Table 1, Panel B, with all figures representing percentage of quality compressions. Performance decreases as the number of compressions without rest increases. As implied by the regression model, all protocols show statistically better performance than continuous compressions, and this result is robust to a Bonferroni correction for the pairwise post hoc tests. Additionally, performance in the 30:10 model also differs significantly from the 100:10 model (t[66]=2.88, p = .032), but there is no statistically significant difference between the 30:10 and 50:10 (t[66]=2.15, p = .213) or between the 50:10 and 100:10 (t[66]=0.74, p = .999).

**Table 1:** Results for compression depth performance (as percent).

Panel A. Mixed effects linear regression model					
	Coef.	SE	df	t	p
Gender	−15.36	16.8	17	−0.91	.374
Age (years)	1.91	2.01	17	0.95	.357
Years certified	−0.37	2.51	17	−0.15	.886
BMI (kg/m^2^)	1.08	1.30	17	0.83	.418
Grip strength (kg)	0.17	0.73	17	0.24	.814
30:10	42.04	6.90	66	6.09	<.001
50:10	27.22	6.90	66	3.94	<.001
100:10	22.13	6.90	66	3.21	.002
**Panel B.** Descriptive statistics of depth percent by protocol					
	Mean	SD	95% CI		
30:10	77.5	28.7	61.8	92.6	
50:10	62.7	37.6	47.0	77.8	
100:10	57.6	36.3	41.9	72.7	
CC	35.5	39.3	19.7	50.5	

The mixed effects linear regression model for the dependent variable of recoil percent was also statistically significant (χ^2^ [8]=52.13, p < .001, Conditional R^2^ = 0.578). Table 2, Panel A, shows that gender, age, BMI, and grip strength were statistically significant for recoil percent in addition to statistically significant differences in CPR quality across the four protocols (F[3, 66]=9.92, p < .001). Furthermore, performance was worse in the CC condition compared to all other conditions. Descriptive statistics of performance quality in Table 2, Panel B, reveal that the 30:10, 50:10, and 100:10 groups were qualitatively similar with substantially overlapping confidence intervals and none of the differences reaching statistical significance, but participants performed considerably better in all cases when compared to the CC protocol.

**Table 2:** Results for recoil performance (as percent).

Panel A. Mixed effects linear regression model					
	Coef.	SE	df	t	p
Gender	24.09	7.28	17	3.31	.004
Age (years)	−2.18	0.87	17	−2.49	.023
Years certified	−0.83	1.08	17	−0.76	.456
BMI (kg/m^2^)	−2.48	0.56	17	−4.40	<.001
Grip strength (kg)	0.70	0.31	17	2.23	.039
30:10	28.13	5.48	66	5.14	<.001
50:10	21.00	5.48	66	3.83	<.001
100:10	21.39	5.48	66	3.91	<.001
**Panel B.** Descriptive statistics of recoil percent by protocol					
	Mean	SD	95% CI		
30:10	89.8	17.0	81.7	99.0	
50:10	82.7	22.5	74.6	91.8	
100:10	83.1	24.4	75.0	92.2	
CC	61.7	36.0	53.6	70.8	

Considering the percent of adequate CPR compression rate as a third quality measure, the overall model was statistically significant (χ^2^ [8]=16.38, p = .037, Conditional R^2^ = 0.401). This model of compression rate is the only one in which age is statistically significant; older participants tended to perform better, as shown in Table 3, Panel A. Descriptive statistics in Table 3, Panel B, show that, once again, performance quality declined as repetitions between breaks increased, even though this difference was statistically significant only in the one case of the comparison between the 50:10 protocol and continuous compressions.

**Table 3:** Results for compression rate performance (as percent).

Panel A. Mixed effects linear regression model					
	Coef.	SE	df	t	p
Gender	−22.76	13.31	17	−1.71	.106
Age (years)	3.69	1.60	17	2.31	.034
Years certified	−2.46	1.98	17	−1.24	.232
BMI (kg/m^2^)	−0.37	1.03	17	−0.36	.722
Grip strength (kg)	−0.14	0.57	17	−0.24	.811
30:10	15.26	9.19	66	1.66	.102
50:10	19.00	9.19	66	2.07	.043
100:10	8.61	9.19	66	0.94	.352
**Panel B.** Descriptive statistics of recoil percent by protocol					
	Mean	SD	95% CI		
30:10	56.8	35.5	41.3	71.3	
50:10	60.5	36.5	45.0	75.0	
100:10	50.1	39.5	34.6	64.7	
CC	41.5	40.5	26.0	56.0	

**Fig 1:**
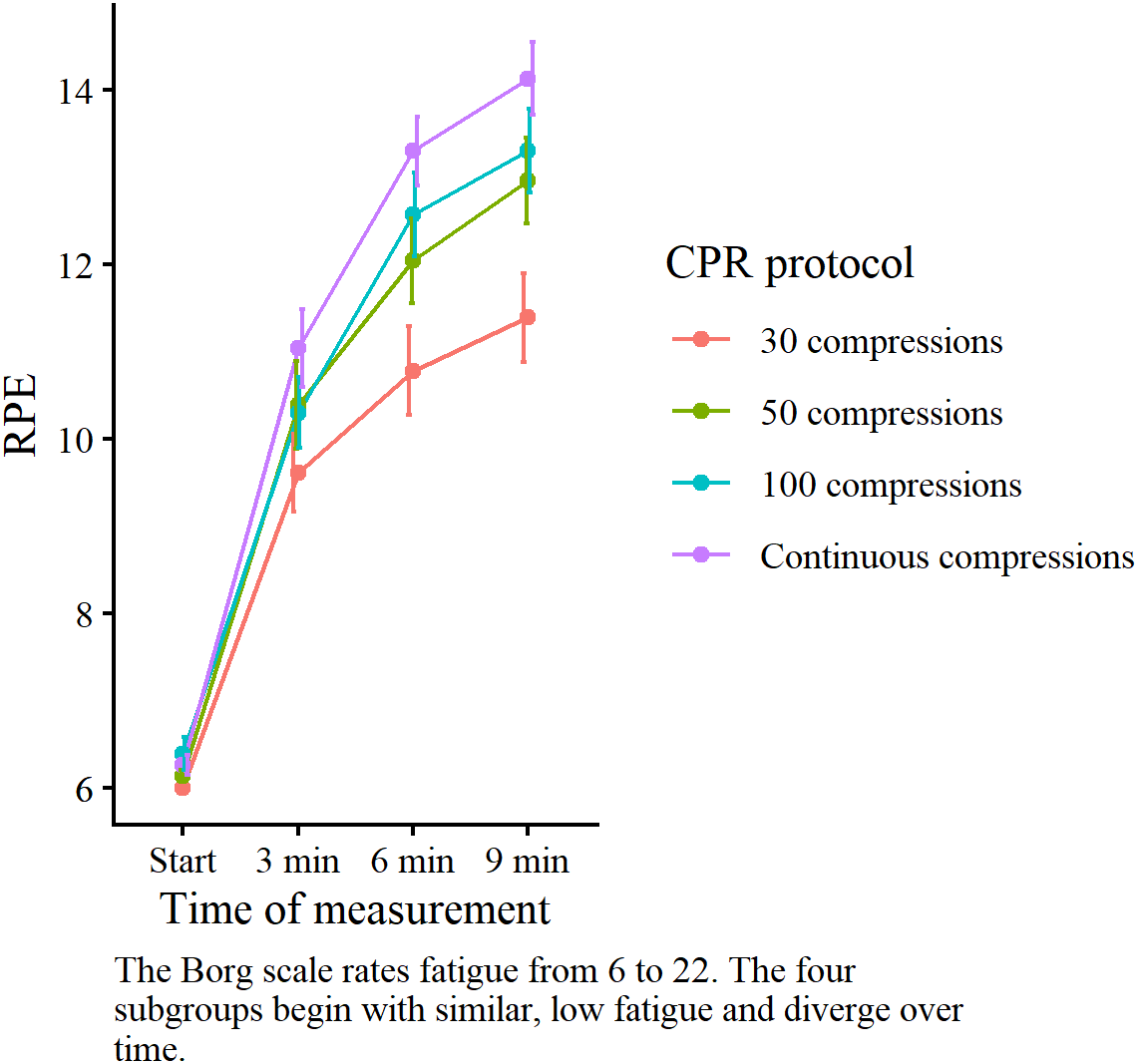
Borg RPE Over Time. The Borg scale rates fatigue from 6 to 22. The four subgroups begin with similar, low fatigue and diverge over time.

The three dependent variables that measured fatigue (i.e., RPE, VO_2_, and heart rate) were modeled with repeated measures ANOVA models with the two within-subjects factors of CPR condition (four levels of 30:10, 50:10, 100:10, and CC) and time (four levels of start, 3, 6, and 9 minutes). In most cases, Mauchly’s test of sphericity was violated, so all reported p-values employ the Greenhouse-Geisser correction. For the repeated measures ANOVA models, the post-hoc power for a large effect size was estimated to be 79.6%. Table 4 provides descriptive statistics of RPE as a measure of fatigue over the two dimensions. Regarding RPE as a subjective measure of fatigue, there was a significant main effect of CPR protocol (F[2.8, 61.1]=11.75, p < .001, η^2^ = .098), indicating that perceived exertion differed across compression protocols. Post-hoc pairwise comparison with a Bonferroni correction showed that in comparison to continuous compressions RPE was significantly higher in all cases, 30:10 (p < .001), 50:10 (p = .037), 100:10 (p = .047). Additionally, the 100:10 condition showed significantly higher RPE than the 30:10 (p = .010) condition. A significant main effect due to time was also observed (F[1.7, 37.0]=243.1, p < .001, η^2^ = .652) with perceived exertion increasing over time (Fig 1). All pairwise comparisons between time points were statistically significant. These main effects are qualified by a significant interaction term (F[5.2, 114.8]=5.22, p < .001, η^2^ = .035). Simple effects analysis revealed that while groups did not differ significantly at the start, differences emerged by 3 minutes and increased in magnitude over time.

**Table 4:** Descriptive statistics of RPE.

RPE	Start	3 min	6 min	9 min
30:10	6.00	9.61	10.78	11.39
	(0.00)	(2.13)	(2.43)	(2.43)
50:10	6.13	10.39	12.04	12.96
	(0.34)	(2.41)	(2.34)	(2.35)
100:10	6.39	10.30	12.56	13.30
	(0.89)	(1.94)	(2.31)	(2.32)
CC	6.26	11.04	13.30	14.13
	(0.54)	(2.14)	(1.89)	(1.98)

Means (SDs) are provided for RPE as a measure of subjective fatigue, collected at four points in time for each of the four CPR protocols. The Borg scale rates fatigue on a scale from 6 to 22.

For the objective fatigue measure of VO_2_, there was a significant main effect due to time (F[1.6, 34.8]=49.73, p < .001, η^2^ = 2.27). Table 5 provides descriptive statistics across the two factors. VO_2_ increases over time, but pairwise follow-up tests reveal that the only statistically significant differences occur between the start rate and the other three observation points. The main effect due to protocol was not statistically significant (F[2.7, 58.4]=2.17, p = .108, η^2^ = .013), nor was there a significant interaction (F[4.6, 100.8]=1.00, p = .421, η^2^ = .007).

**Table 5:** Descriptive statistics of VO_2_ (mL/kg/min).

VO2	Start	3 min	6 min	9 min
30:10	5.09	10.75	11.65	10.88
	(2.67)	(5.65)	(6.61)	(4.90)
50:10	4.46	12.53	11.88	11.57
	(3.18)	(6.21)	(6.95)	(6.18)
100:10	5.37	13.36	13.43	12.96
	(2.92)	(6.64)	(6.63)	(7.04)
CC	6.05	13.32	12.06	11.46
	(5.47)	(5.94)	(5.29)	(5.76)

Means (SDs) are provided for VO2 as a measure of objective fatigue, collected at four points in time for each of the four CPR protocols.

Finally, for heart rate as an objective measure of fatigue, the time dimension is again statistically significant with a large effect size (F[1.8, 39.7]=108.78, p < .001, η^2^ = .288), but the comparison across CPR protocol is not statistically significant with a small effect size (F[1.8, 39.9]=2.39, p = .109, η^2^ = .012). Table 6 provides descriptive statistics across the two dimensions of time and CPR protocol. Heart rate increases over time, but as with VO_2_, the increase occurs from the starting level to the active period, based on Bonferroni-corrected post hoc tests. The three later measurements do not differ from each other to a statistically significant extent.

**Table 6:** Descriptive statistics of heart rate (bpm).

HR	Start	3 min	6 min	9 min
30:10	101.59	126.48	125.07	122.39
	(20.78)	(23.73)	(20.65)	(18.52)
50:10	102.02	125.02	129.26	128.30
	(22.01)	(17.36)	(19.01)	(17.38)
100:10	92.48	124.33	127.85	127.30
	(20.36)	(17.80)	(18.66)	(18.21)
CC	102.35	127.76	130.39	133.26
	(15.01)	(19.59)	(21.82)	(16.44)

Means (SDs) are provided for heart rate as a measure of objective fatigue, collected at four points in time for each of the four CPR protocols.

## Discussion

This study advances CPR research by delivering a comprehensive analysis of rescuer fatigue, integrating metabolic, subjective, and performance metrics to help determine optimal compression-to-rest ratios that enhance rescuer performance and, by implication, patient outcomes. The study assessed subjective fatigue using the Rating of Perceived Exertion (RPE) alongside two objective measures of fatigue: VO_2_ and HR. Findings echoed those observed in many physical tasks, revealing that both VO_2_ and HR initially increased before plateauing across all four conditions, indicating that participants reached a steady state, consistent with previous results of Shin et al. [6] and Ock et al. [21] Furthermore, when comparing compression-only CPR to the 30:2 method, compression-only CPR has been associated with greater increases in HR [15] and muscle fatigue [28], as well as significant decreases in compression force [7]. These results may stem, in part, from the increased number of compressions with the continuous protocol. Additionally, there is evidence suggesting that an elevated HR correlates with reduced accuracy in chest compressions [29].

A rescuer’s weight and strength have previously been shown to be important factors influencing CPR performance. Ock et al. [21] reported that greater muscle strength, measured by maximum hand grip contraction, correlated with higher-quality chest compressions. Similarly, Lopez-Gonzalez et al. [18] reported that participants with lower strength delivered fewer effective compressions. However, the only CPR component significantly associated with strength in the present study was chest recoil. While many researchers have indicated that individuals with higher body mass index (BMI) perform better than lighter participants [18,30,31], findings here suggest that BMI influences only the percentage of full recoil achieved.

The lack of focus on chest recoil in CPR research is a significant limitation, as full recoil is crucial for creating negative pressure in the thoracic cavity, enhancing venous return and blood flow to the heart [32–34]. Incomplete recoil increases intrathoracic pressure, reducing compression effectiveness and compromising patient outcomes, which undermines the development of effective CPR guidelines. The results here indicate that as the number of uninterrupted compressions increases, the proportion of compressions achieving full chest recoil decreases. Given the essential role of chest wall decompression in CPR efficacy, further research is necessary to address this critical area.

Objective fatigue measures provide insights into CPR’s physiological aspects, but subjective measurements may better indicate CPR quality. The present study found that subjective fatigue increased during the 8:59 CPR sessions and correlated more strongly with performance than objective measures. Ock et al. [21] similarly reported rising RPE during five minutes of compression-only CPR, which were linked to fewer adequate compressions. Higher RPE values have also been associated with continuous compressions compared to the 30:2 method, indicating decreased compression quality [7]. While other studies have produced mixed results on subjective fatigue, their short durations do not reflect the typical demands on rescuers [6,15,17]. In the longer time periods employed in this study, compression-only CPR resulted in greater subjective fatigue.

The statistical significance of self-reported (subjective) fatigue but not the objective measures of fatigue across the different CPR protocols aligns with the psychobiological model of endurance performance [35]. This model argues that perceived fatigue decreases physical performance, even though objective, physiological measures may not reflect a similar level of fatigue [36]. Data from a CPR task follow this pattern, reporting relatively low physiological demands but high RPE [37], which is consistent with the data in the present study and with the idea that perceived fatigue could be a factor in the consistent observation of diminished CPR quality over time [7,15].

One aspect that distinguishes this study from previous research is the examination of four distinct compression-only CPR conditions. The critical factor for successful cardiac arrest outcomes is the number of effective chest compressions delivered, rather than the total compressions attempted [33,34]. This consideration is especially important for continuous compression protocols, which, while yielding more compressions, are associated with fatigue and reduced CPR quality. Several studies have reported similar findings, noting declines in compression quality over time during compression-only CPR compared to various compression-to-ventilation (C:V) ratios [5,7,15]. As the number of compressions increases, CPR quality decreases and fatigue increases. The findings are meaningful because they demonstrate that the implementation of a brief rest period might help preserve a lay rescuer’s ability to provide care.

### Limitations

Generalization of the findings here is limited by the convenience nature of the demographic. More specifically, the age range is relatively young which affects generalizability to older or more diverse CPR providers. Additionally, while participants were lay rescuers, they had all earned CPR certification, so the results cannot be extrapolated to real-world bystanders, who may be older, less fit, and/or lack CPR training. The use of a manikin provides valuable data in a controlled environment, but it does not fully reflect human physiology or allow for assessment of clinical outcomes, which would both require empirical data to be collected from national medical statistics. Another limitation of the study is that the relationship between the measures of fatigue and CPR performance quality are correlational, not causal. However, this study comprehensively combines measures of objective and subjective fatigue with multiple measures of CPR performance quality, and the findings align with previous research to suggest that continuous chest compressions result in diminished CPR quality. In particular, participants were unable to sustain performance quality over the 9-minute time period that is necessary, on average, for an ambulance to arrive. Before any proposal can be made to update CPR protocol recommendations, future work will also need to weigh the tradeoffs in chest compression quantity and quality that are implicit in the consideration of fatigue and rest periods.

## Conclusion

Ongoing revisions to the AHA Guidelines are crucial for improving survival rates from sudden cardiac arrest. The findings here suggest that as the number of compressions without rest increases, CPR performance declines. While physiological measures show minimal increases in objective fatigue, subjective fatigue data may be more indicative of CPR performance. Therefore, the results do not completely support a recommendation for continuous chest compressions without a break. We advocate for further research to investigate the benefits of a 10-second rest during compression-only CPR to enhance chest compression quality. Rest periods could potentially enable rescuers to maintain quality care and improve outcomes in out-of-hospital cardiac arrest situations. This study demonstrates that rescuer fatigue adversely affects chest compression quality over time, which raises an important question that merits further research to maximize patient benefits from compression only CPR protocols.

## Supporting information

S1 Data Anonymized Data Set. (XLSX)
